# Supplementary figures and images for: Engineering the green algae Chlamydomonas incerta for recombinant protein production
Source: PLoS One. 2025 Apr 16;20(4):e0321071. doi: 10.1371/journal.pone.0321071 (PMC12002436; doi:10.1371/journal.pone.0321071)

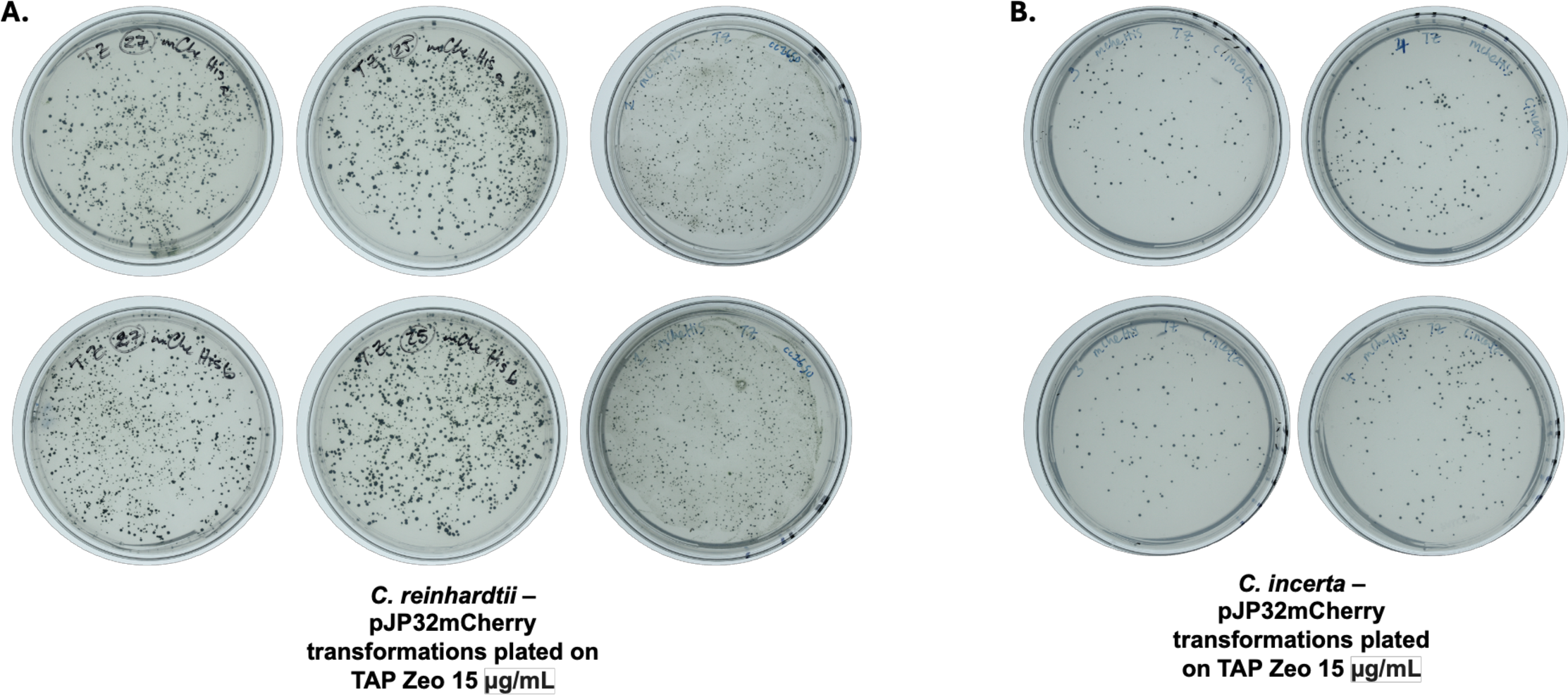

Supplement: S1 Fig — The mCherry secretion vector, pJP32mCherry, was transformed into A) C. reinhardtii and B) C. incerta. Triplicate transformations were performed for both species, and transformants were spread onto two plates (paired vertically). However, only two transformations for C. incerta generated an adequate amount of colonies, so the third transformation is not shown. (TIF) [file pone.0321071.s001.tif]

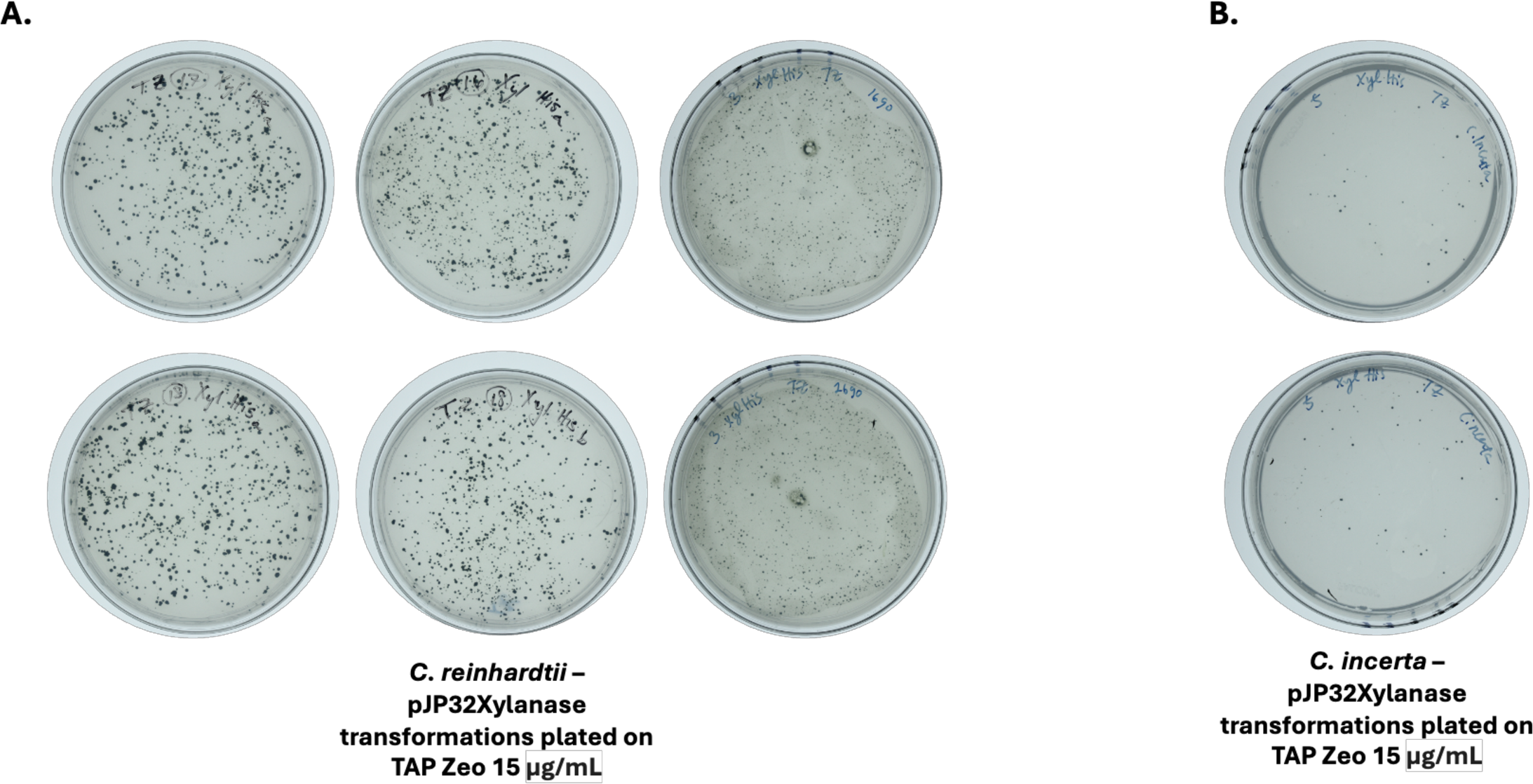

Supplement: S2 Fig — The xylanase secretion vector, pJP32Xylanase, was transformed into A) C. reinhardtii and B) C. incerta. Triplicate transformations were performed for both species, and transformants were spread onto two plates (paired vertically). However, only one transformation for C. incerta generated an adequate amount of colonies, so the other two transformations are not shown. (TIF) [file pone.0321071.s002.tif]

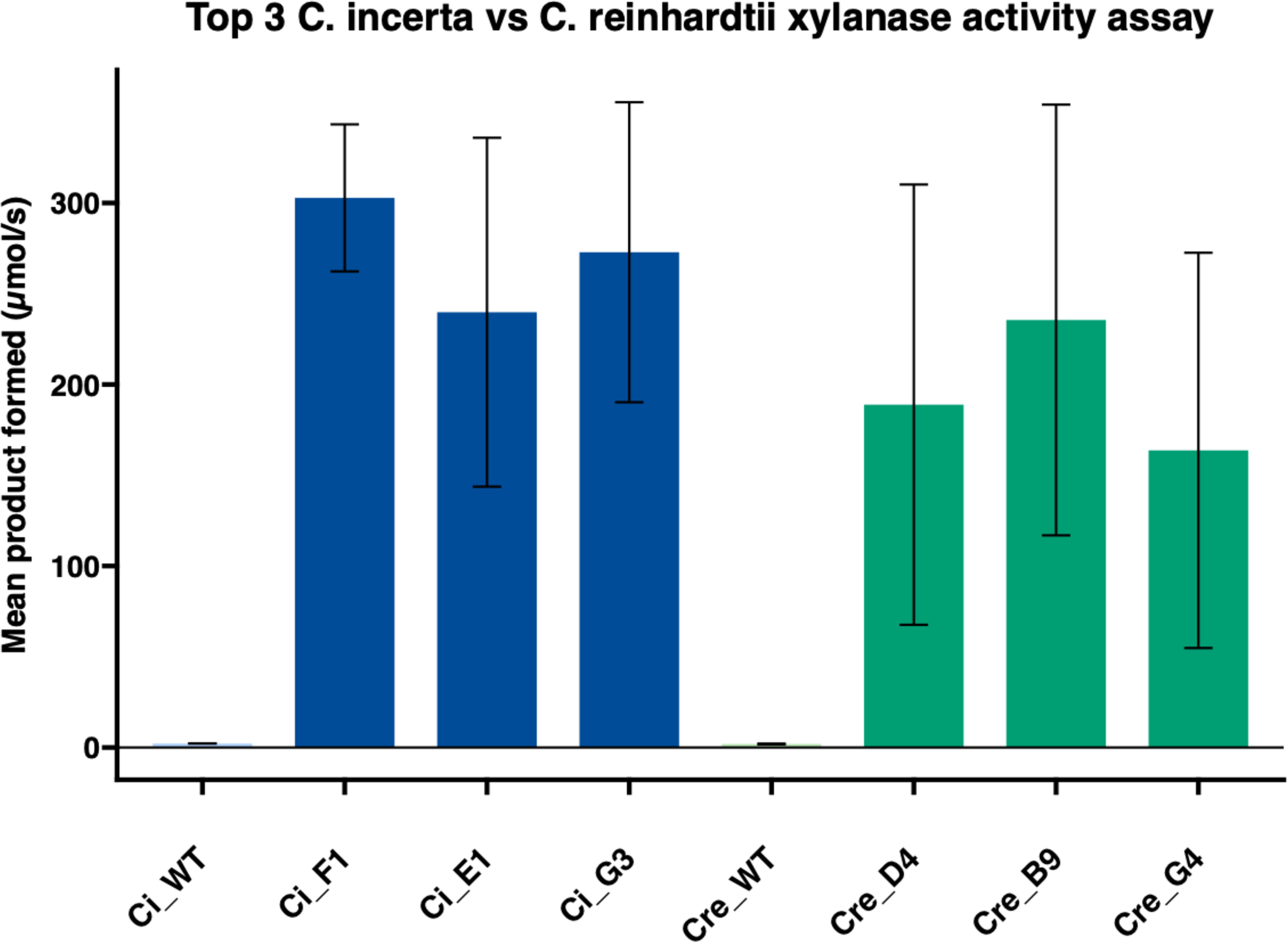

Supplement: S3 Fig — Hydrolysis of the fluorogenic substrate 6,8-difluoro-4-methylumbelliferyl β-d-xylobioside (DiFMUX2) by xylanase led to increased fluorescence at an excitation wavelength of 385 nm and emission wavelength of 455 nm over time. The F1, E1, and G3 strains of transgenic C. incerta expressing xylanase formed 302.799 µmol/s, 239.806 µmol/s, and 272.852 µmol/s of product, respectively. The D4, B9, and G3 strains of transgenic C. reinhardtii expressing xylanase formed 188.873 µmol/s, 235.523 µmol/s, and 163.695 µmol/s of product, respectively. The C. incerta and C. reinhardtii wild types formed 2.219 µmol/s and 1.918 µmol/s of product, respectively. (TIF) [file pone.0321071.s003.tif]

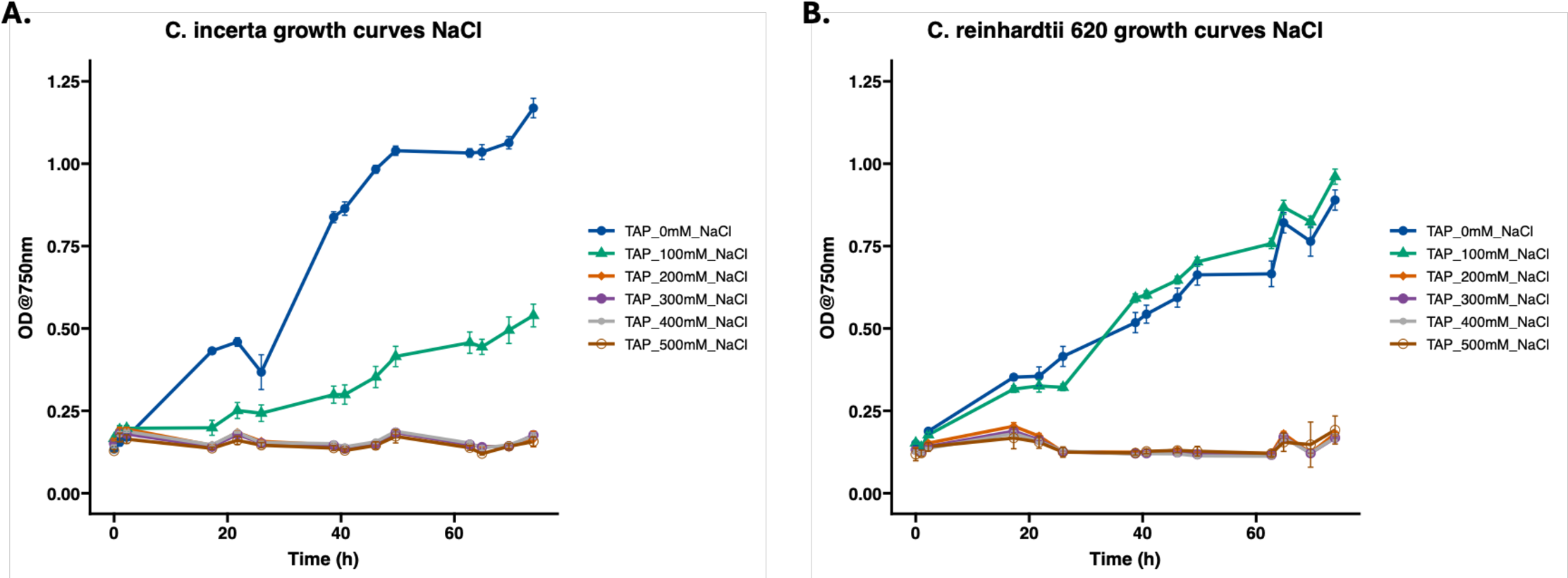

Supplement: S4 Fig — A) The C. incerta wild type was grown in TAP media containing 0 mM, 100 mM, 200 mM, 300 mM, 400 mM, and 500 mM NaCl. Absorbance readings at 750 nm were measured using the Infinite® M200 PRO plate reader (Tecan, Männedorf, Switzerland) over approximately 73 hours, and the readings represent the average of biological quadruplicates. B) The C. reinhardtii wild type was grown in TAP media containing 0 mM, 100 mM, 200 mM, 300 mM, 400 mM, and 500 mM NaCl. Absorbance readings at 750 nm were measured using the Infinite® M200 PRO plate reader (Tecan, Männedorf, Switzerland) over approximately 73 hours, and the readings represent the average of biological quadruplicates. (TIF) [file pone.0321071.s004.tif]

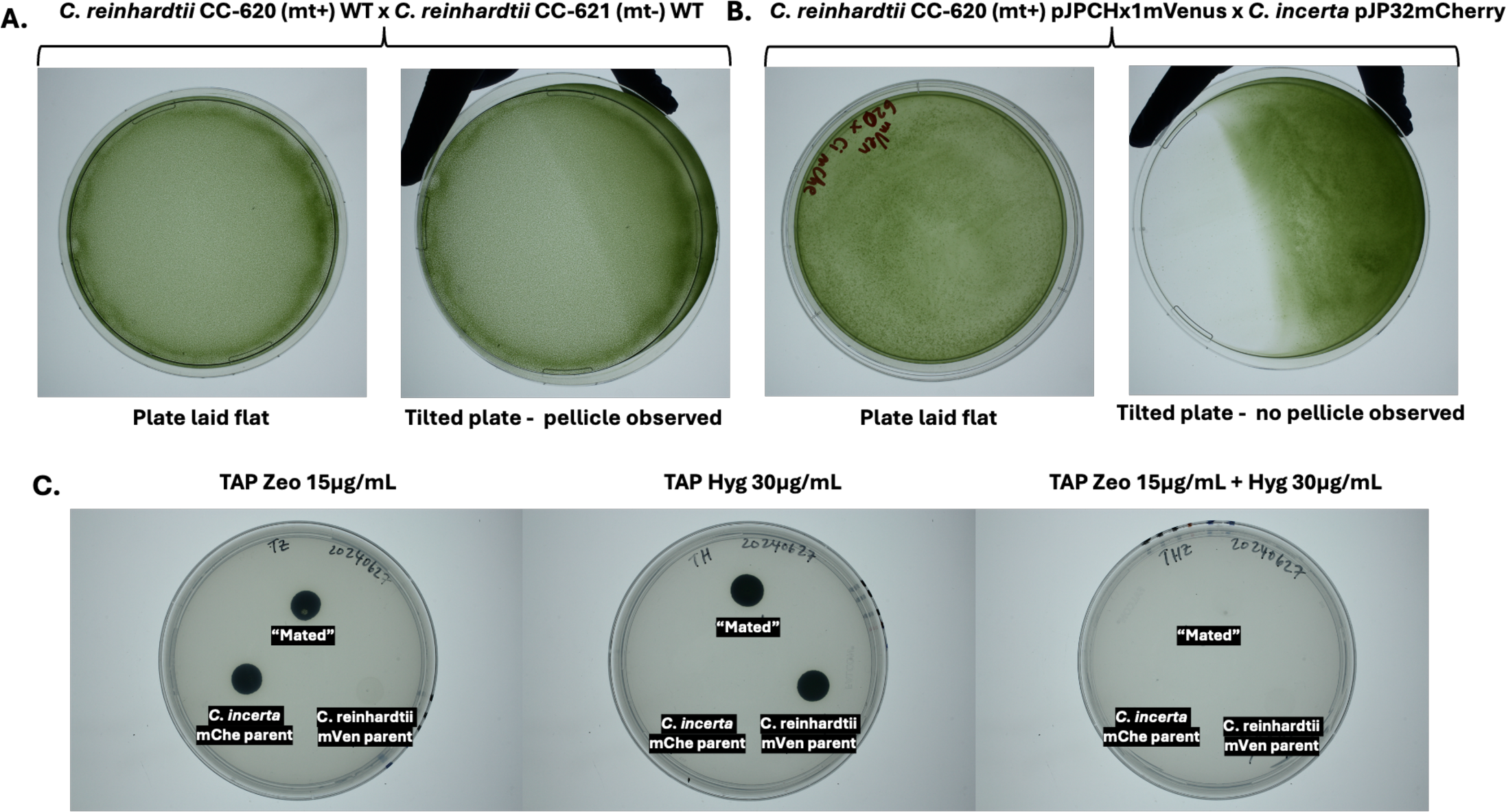

Supplement: S5 Fig — A) C. reinhardtii CC-620 (mt+) wild type and C. reinhardtii CC-621 (mt-) wild type were mated, and a pellicle phenotype was observed after 12–16 hours, indicating success of intraspecies mating. B) Transgenic C. reinhardtii CC-620 (mt+) pJPCHx1mVenus and transgenic C. incerta pJP32mCherry, and a pellicle phenotype was not observed after 12–16 hours, indicating that interspecies mating is not feasible. C) The mixed cells that did not formed a pellicle from B, along with the C. reinhardtii pJPCHx1mVenus and C. incerta pJP32 parents were plated onto 3 types of plates: TAP agar plates containing zeocin 15 µg/mL, TAP agar plates containing hygromycin B 30 µg/mL, and TAP agar plates containing both zeocin 15 µg/mL and hygromycin 30 µg/mL. (TIF) [file pone.0321071.s005.tif]

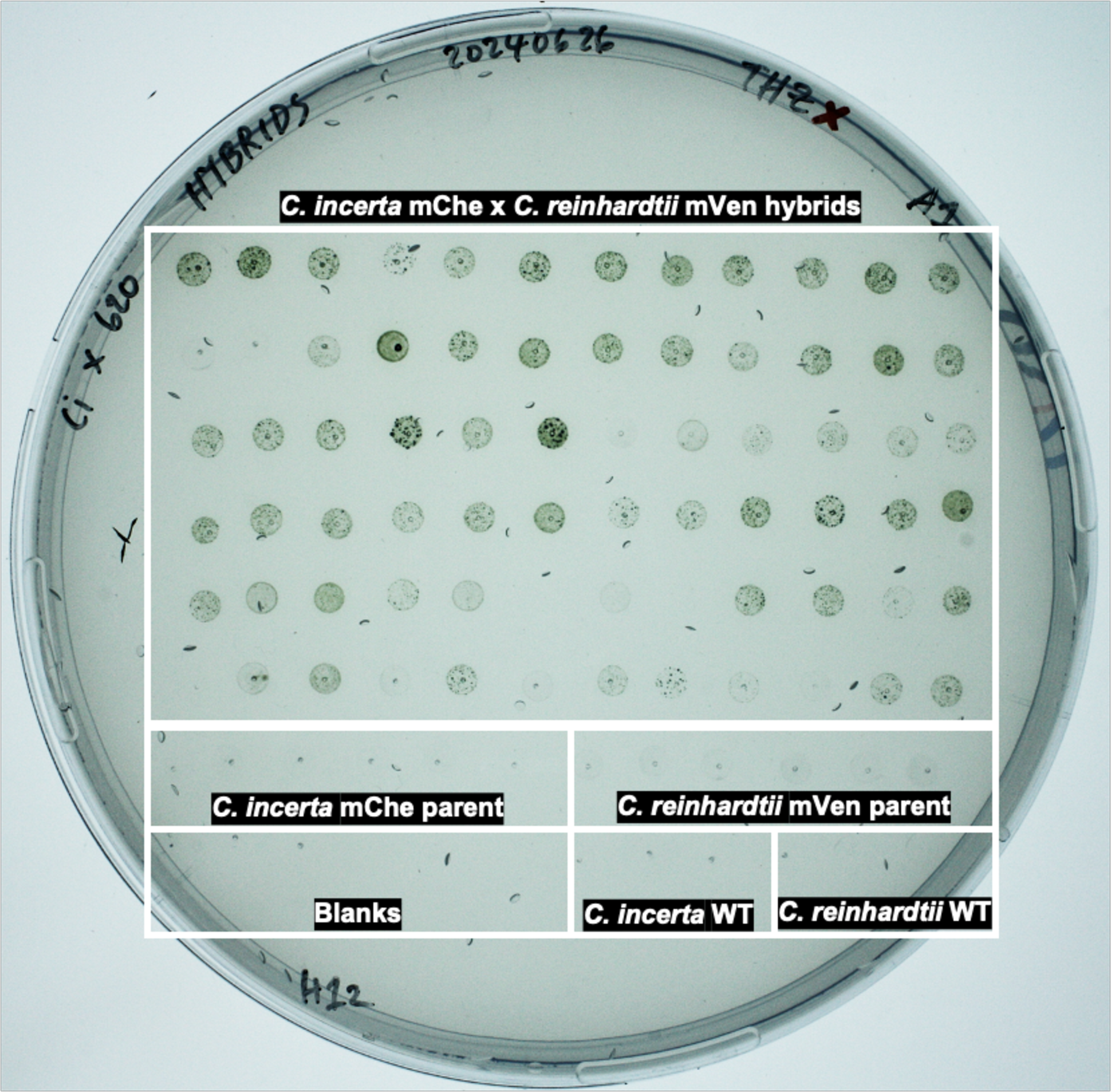

Supplement: S6 Fig — Potential hybrid colonies from the transformation plate were picked into 96-well plates containing TAP media and grown for 7 days. Replica TAP agar plate containing zeocin 15 µg/mL and hygromycin 30 µg/mL was made to screen for stable hybrids. (TIF) [file pone.0321071.s006.tif]

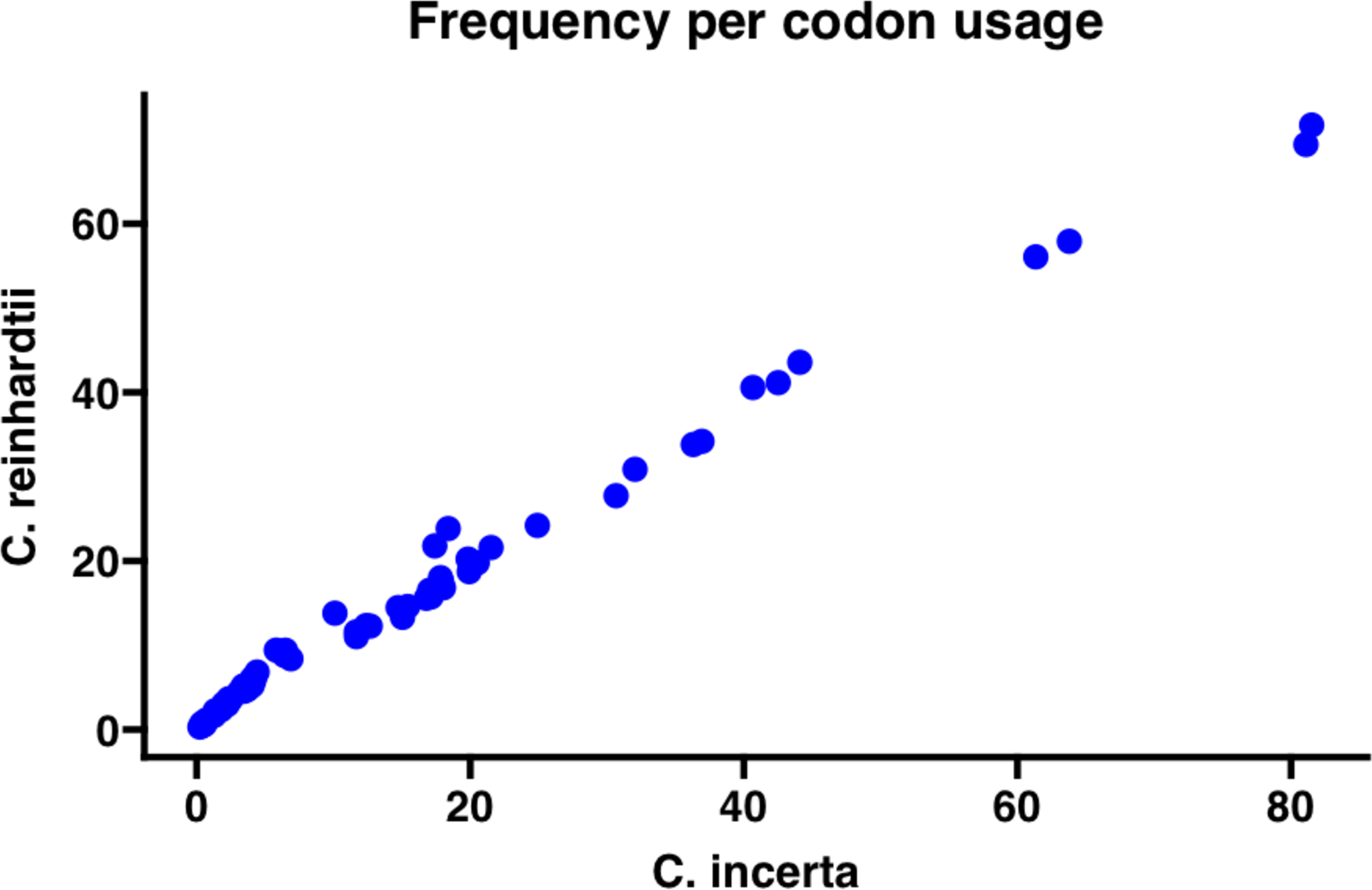

Supplement: S7 Fig — The scatter plot depicts the frequency of each codon’s usage in C. incerta (x-axis) against C. reinhardtii (y-axis). A positive 1:1 correlation in codon usage patterns is observed between the two species, suggesting similarities in their codon preferences despite genomic differences. (TIF) [file pone.0321071.s007.tif]

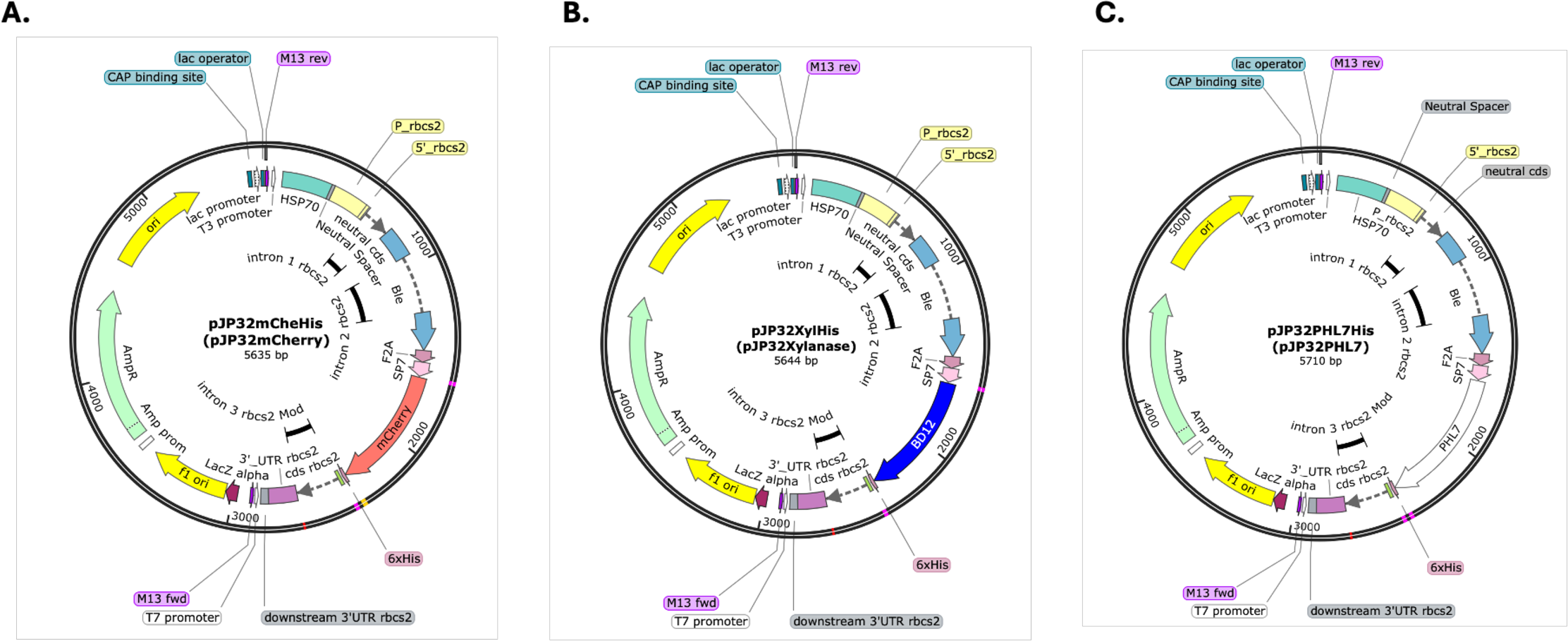

Supplement: S8 Fig — Plasmid maps for A) pJP32mCheHis (denoted as pJP32mCherry in the publication), B) pJP32XylHis (pJP32Xylanase), and C) pJP32PHL7His (pJP32PHL7) using SnapGene (GSL Biotech LLC, San Diego, CA, USA). (TIF) [file pone.0321071.s008.tif]

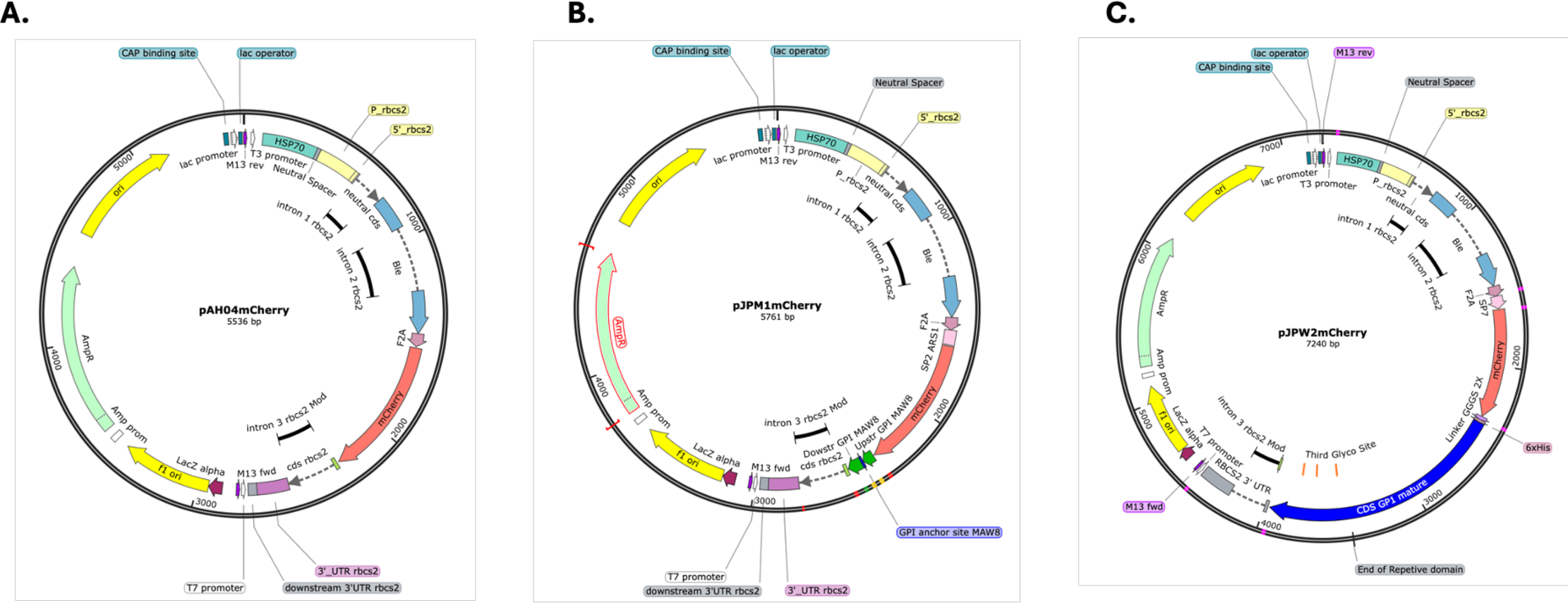

Supplement: S9 Fig — Plasmid maps for A) pAH04mCherry, an mCherry cytosolic expression vector, B) pJPM1mCherry, an mCherry cell membrane expression vector, and C) pJPW2mCherry, an mCherry cell wall expression vector, using SnapGene (GSL Biotech LLC, San Diego, CA, USA). (TIF) [file pone.0321071.s009.tif]

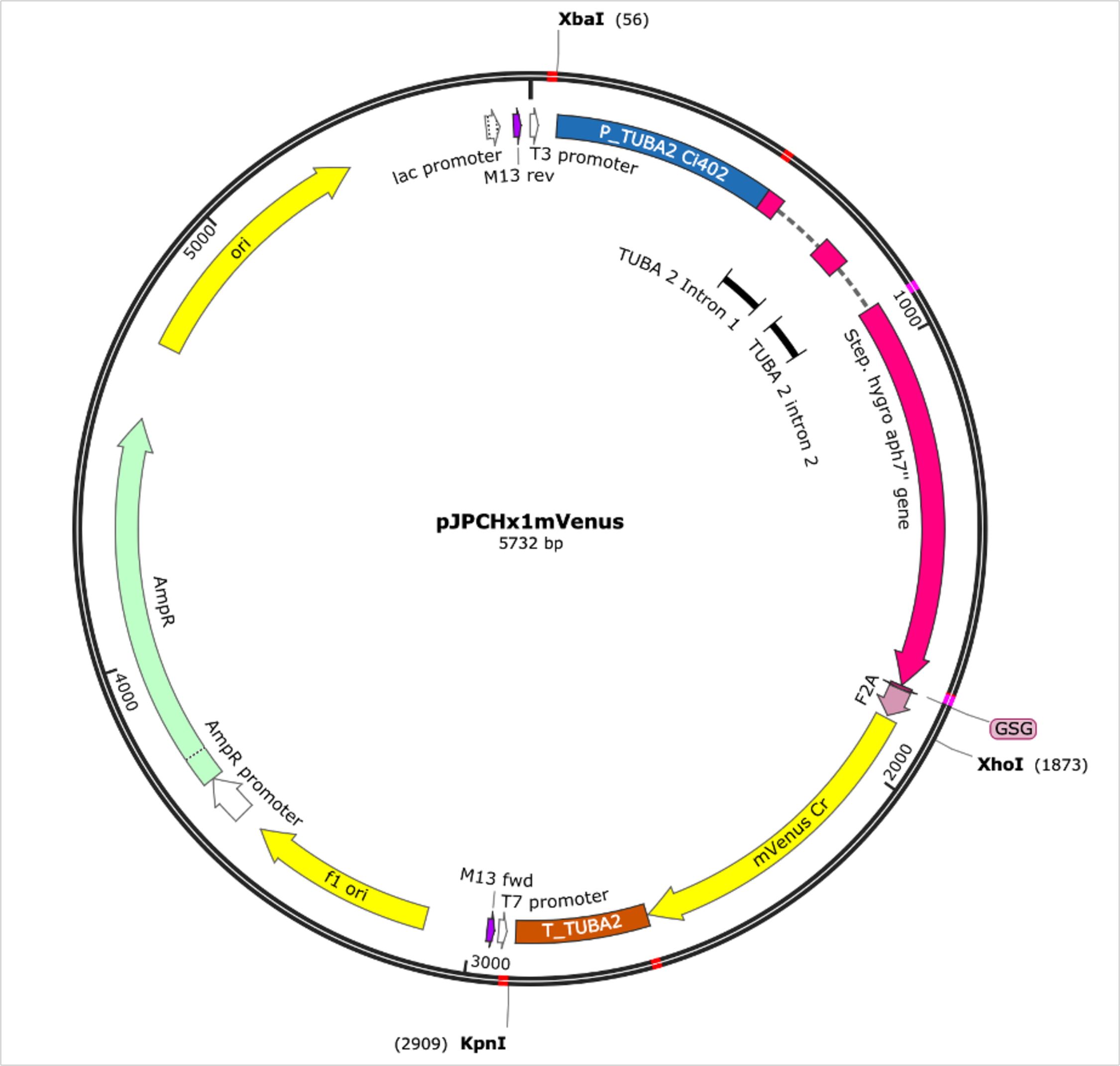

Supplement: S10 Fig — Plasmid map for pJPCHx1mVenus, an mVenus cytosolic expression vector containing the hygromycin B antibiotic resistance gene, using SnapGene (GSL Biotech LLC, San Diego, CA, USA). (TIF) [file pone.0321071.s010.tif]

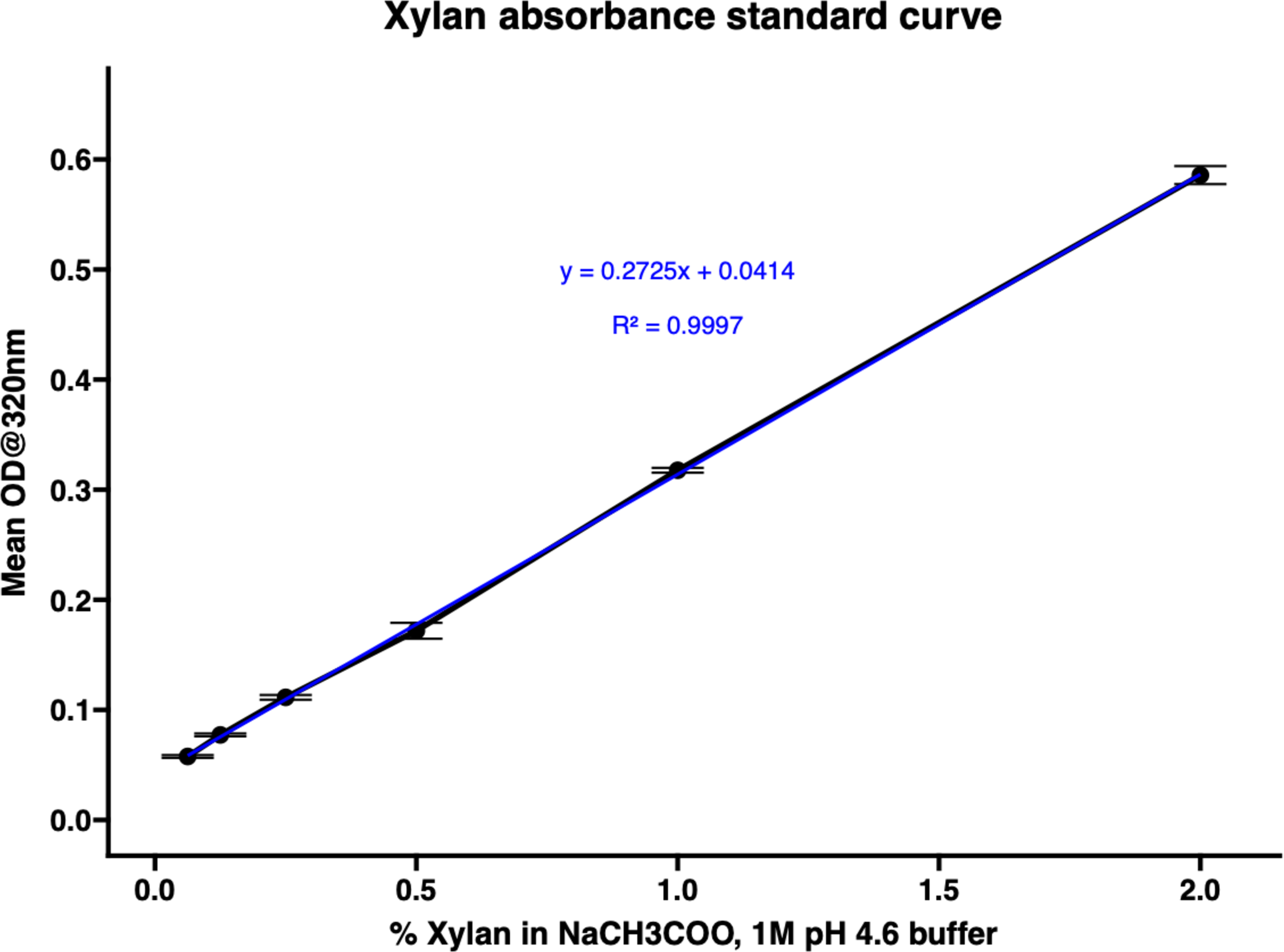

Supplement: S11 Fig — The xylan absorbance standard curve was created using varying concentrations of Xylan from Corn Core (0%, 0.0625%, 0.125%, 0.25%, 0.5%, 1%, and 2% w/v) in sodium acetate buffer (1M, pH 4.5). Absorbance at 320 nm were measured in the 96-well UV-StarⓇ microplates (Greiner Bio-One, Kremsmünster, Austria) using the Infinite® M200 PRO plate reader (Tecan, Männedorf, Switzerland). The standard curve was fitted with a linear regression equation y = mx + b, where m is the slope and b is the y-intercept (equation: y = 0.2727x + 0.0414; R2 = 0.9997). (TIF) [file pone.0321071.s011.tif]

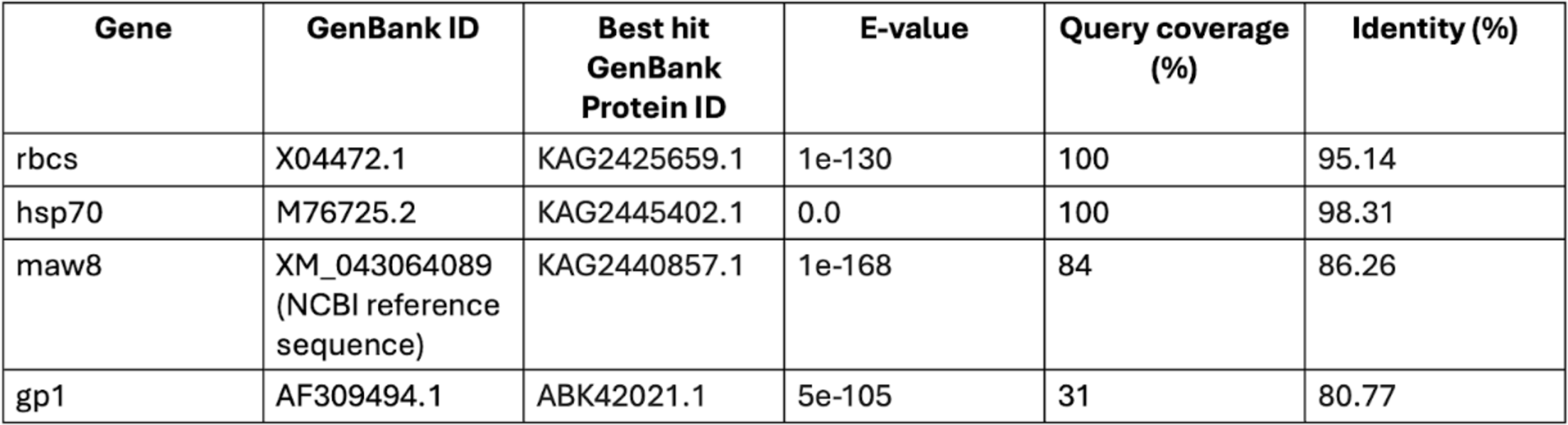

Supplement: S1 Table — The table shows the GenBank IDs for the queried genes, the best hit GenBank protein ID, the E-value indicating the statistical significance of the match, the query coverage percentage, and the percentage identity of the aligned sequences. (TIF) [file pone.0321071.s012.tif]
