# Supplementary material for: Engineering the green algae Chlamydomonas incerta for recombinant protein production
Source: PLoS One. 2025 Apr 16;20(4):e0321071. doi: 10.1371/journal.pone.0321071 (PMC12002436; doi:10.1371/journal.pone.0321071)
Supplement: Attachment — Submitted filename: Response to Reviewers.pdf [file pone.0321071.s014.pdf]

Response to reviewers

Journal Requirements:

1. When submitting your revision, we need you to address these additional requirements.

Please ensure that your manuscript meets PLOS ONE's style requirements, including those for file naming. The PLOS ONE style templates can be found at https://journals.plos.org/plosone/s/file?id=wjVg/PLOSOne_formatting_sample_main_body.pdf and https://journals.plos.org/plosone/s/file?id=ba62/PLOSOne_formatting_sample_title_authors_affiliations.pdf

Authors response:

We reformatted the manuscript to meet PLOS ONE's style requirements.

2. Thank you for stating in your Funding Statement:
This material is based upon work supported by the U.S. Department of Energy's Office of Energy Efficiency and Renewable Energy (EERE) under the APEX award number DE-EE0009671. This material was also supported by the S10OD030505 and NINDS P30NS047101 grants from the UCSD microscopy core.

Please provide an amended statement that declares *all* the funding or sources of support (whether external or internal to your organization) received during this study, as detailed online in our guide for authors at http://journals.plos.org/plosone/s/submit-now. Please also include the statement "There was no additional external funding received for this study." in your updated Funding Statement.
Please include your amended Funding Statement within your cover letter. We will change the online submission form on your behalf.

Authors response:

We adjusted our Funding Statement to declare all funding sources.

"This material is based upon work supported by the U.S. Department of Energy's Office of Energy Efficiency and Renewable Energy (EERE) under the APEX award number DE-EE0009671. This material was also supported by the S10OD030505 and NINDS P30NS047101 grants from the UCSD microscopy core. There was no additional external funding received for this study. The funder provided support in the form of salaries for authors but did not have any additional role in the study design, data collection and analysis, decision to publish, or preparation of the manuscript. The specific roles of authors are articulated in the 'Author Contributions' section. This does not alter our adherence to PLOS ONE policies on sharing data and materials. The remaining authors declare no competing financial or commercial interests."

3. Thank you for stating the following in the Acknowledgments Section of your manuscript:
The authors gratefully acknowledge the support from the United States Department of Energy, the California Center for Algae Biotechnology, and the University of California San Diego. They also acknowledge the support of imaging specialist Marcy Erb, Ph.D. for her contribution in the microscopy core.

We note that you have provided funding information that is not currently declared in your Funding Statement. However, funding information should not appear in the Acknowledgments section or other areas of your manuscript. We will only publish funding information present in the Funding Statement section of the online submission form.
Please remove any funding-related text from the manuscript and let us know how you would like to update your Funding Statement. Currently, your Funding Statement reads as follows:
This material is based upon work supported by the U.S. Department of Energy's Office of Energy Efficiency and Renewable Energy (EERE) under the APEX award number DE-EE0009671. This material was also supported by the S10OD030505 and NINDS P30NS047101 grants from the UCSD microscopy core.

Please include your amended statements within your cover letter; we will change the online submission form on your behalf.

Authors response:

We updated the text and added it to the cover letter as suggested.

Funding Statement:

"This material is based upon work supported by the U.S. Department of Energy's Office of Energy Efficiency and Renewable Energy (EERE) under the APEX award number DE-EE0009671. This material was also supported by the S10OD030505 and NINDS P30NS047101 grants from the UCSD microscopy core. There was no additional external funding received for this study. The funder provided support in the form of salaries for authors but did not have any additional role in the study design, data collection and analysis, decision to publish, or preparation of the manuscript. The specific roles of authors are articulated in the 'Author Contributions' section. This does not alter our adherence to PLOS ONE policies on sharing data and materials. The remaining authors declare no competing financial or commercial interests."

Acknowledgments:

"The authors gratefully acknowledge the support of imaging specialist Marcy Erb, Ph.D. for her contribution in the microscopy core."

4. Thank you for stating the following in the Competing Interests section:
Stephen Mayfield was a founding of and holds an equity stake in Algenesis Inc, a company that

could potentially benefit from this work. The remaining authors declare that the research was conducted in the absence of any commercial or financial relationships that could be construed as a potential conflict of interest.

We note that one or more of the authors are employed by a commercial company: Algenesis Inc,.

a. Please provide an amended Funding Statement declaring this commercial affiliation, as well as a statement regarding the Role of Funders in your study. If the funding organization did not play a role in the study design, data collection and analysis, decision to publish, or preparation of the manuscript and only provided financial support in the form of authors' salaries and/or research materials, please review your statements relating to the author contributions, and ensure you have specifically and accurately indicated the role(s) that these authors had in your study. You can update author roles in the Author Contributions section of the online submission form.

Please also include the following statement within your amended Funding Statement.
“The funder provided support in the form of salaries for authors, but did not have any additional role in the study design, data collection and analysis, decision to publish, or preparation of the manuscript. The specific roles of these authors are articulated in the ‘author contributions’ section.”
If your commercial affiliation did play a role in your study, please state and explain this role within your updated Funding Statement.

Authors response:

We revised the Funding Statement:
“This material is based upon work supported by the U.S. Department of Energy’s Office of Energy Efficiency and Renewable Energy (EERE) under the APEX award number DE-EE0009671. This material was also supported by the S10OD030505 and NINDS P30NS047101 grants from the UCSD microscopy core. There was no additional external funding received for this study. The funder provided support in the form of salaries for authors but did not have any additional role in the study design, data collection and analysis, decision to publish, or preparation of the manuscript. The specific roles of authors are articulated in the ‘Author Contributions’ section. This does not alter our adherence to PLOS ONE policies on sharing data and materials. The remaining authors declare no competing financial or commercial interests.”

b. Please also provide an updated Competing Interests Statement declaring this commercial affiliation along with any other relevant declarations relating to employment, consultancy, patents, products in development, or marketed products, etc.

Within your Competing Interests Statement, please confirm that this commercial affiliation does not alter your adherence to all PLOS ONE policies on sharing data and materials by including

the following statement: "This does not alter our adherence to PLOS ONE policies on sharing data and materials.” (as detailed online in our guide for authors http://journals.plos.org/plosone/s/competing-interests) . If this adherence statement is not accurate and there are restrictions on sharing of data and/or materials, please state these. Please note that we cannot proceed with consideration of your article until this information has been declared.

Please include both an updated Funding Statement and Competing Interests Statement in your cover letter. We will change the online submission form on your behalf.

Authors response:

We updated the Funding statement and Competing interests statement:
“SM was a founding of and holds an equity stake in Algenesis Materials, a company that could potentially benefit from this work. The remaining authors declare that the research was conducted in the absence of any commercial or financial relationships that could be construed as a potential conflict of interest. This does not alter our adherence to PLOS ONE policies on sharing data and materials.”

5. Please include captions for your Supporting Information files at the end of your manuscript, and update any in-text citations to match accordingly. Please see our Supporting Information guidelines for more information: http://journals.plos.org/plosone/s/supporting-information.

Authors response:

We included the captions to our Supporting Information files at the end of the manuscript and updated in-text citations.

6. PLOS ONE now requires that authors provide the original uncropped and unadjusted images underlying all blot or gel results reported in a submission’s figures or Supporting Information files. This policy and the journal’s other requirements for blot/gel reporting and figure preparation are described in detail at https://journals.plos.org/plosone/s/figures#loc-blot-and-gel-reporting-requirements and https://journals.plos.org/plosone/s/figures#loc-preparing-figures-from-image-files. When you submit your revised manuscript, please ensure that your figures adhere fully to these guidelines and provide the original underlying images for all blot or gel data reported in your submission. See the following link for instructions on providing the original image data: https://journals.plos.org/plosone/s/figures#loc-original-images-for-blots-and-gels.
In your cover letter, please note whether your blot/gel image data are in Supporting Information

or posted at a public data repository, provide the repository URL if relevant, and provide specific details as to which raw blot/gel images, if any, are not available. Email us at plosone@plos.org if you have any questions.

Authors response:

The original uncropped and unadjusted images underlying all blot or gel results have been compiled into a single .pdf file entitled “Original blot and gel images”.

7. Please review your reference list to ensure that it is complete and correct. If you have cited papers that have been retracted, please include the rationale for doing so in the manuscript text, or remove these references and replace them with relevant current references. Any changes to the reference list should be mentioned in the rebuttal letter that accompanies your revised manuscript. If you need to cite a retracted article, indicate the article’s retracted status in the References list and also include a citation and full reference for the retraction notice.

Authors response:

The reference list is complete and correct.

Additional Editor Comments:

I agree withe the reviewer and the easiest approach will be to revise the title. Please return the manuscript and include a detailed response of how you chose the new title.

Authors response:

We elected to change the title to “Engineering the green algae *Chlamydomonas incerta* for recombinant protein production” to reflect the strain engineering focus of our work.

Reviewers' comments:

Reviewer's Responses to Questions

Comments to the Author

1. Is the manuscript technically sound, and do the data support the conclusions?

The manuscript must describe a technically sound piece of scientific research with data that

supports the conclusions. Experiments must have been conducted rigorously, with appropriate controls, replication, and sample sizes. The conclusions must be drawn appropriately based on the data presented.

Reviewer #1: Yes

---

2. Has the statistical analysis been performed appropriately and rigorously?

Reviewer #1: Yes

---

3. Have the authors made all data underlying the findings in their manuscript fully available?

The PLOS Data policy requires authors to make all data underlying the findings described in their manuscript fully available without restriction, with rare exception (please refer to the Data Availability Statement in the manuscript PDF file). The data should be provided as part of the manuscript or its supporting information, or deposited to a public repository. For example, in addition to summary statistics, the data points behind means, medians and variance measures should be available. If there are restrictions on publicly sharing data—e.g. participant privacy or use of data from a third party—those must be specified.

Reviewer #1: Yes

---

4. Is the manuscript presented in an intelligible fashion and written in standard English?

PLOS ONE does not copyedit accepted manuscripts, so the language in submitted articles must be clear, correct, and unambiguous. Any typographical or grammatical errors should be corrected at revision, so please note any specific errors here.

Reviewer #1: Yes

---

5. Review Comments to the Author

Please use the space provided to explain your answers to the questions above. You may also include additional comments for the author, including concerns about dual publication, research ethics, or publication ethics. (Please upload your review as an attachment if it exceeds 20,000 characters)

Reviewer #1: The manuscript by Kang et al. describes strain engineering work comparing protein expression titers in C. incerta and C. reinhardtii. A 3-fold increase in transgene fluorescence and protein titer was shown for one gene construction in C. incerta relative to C.

reinhardtii. Other constructs provided similar results between the strains. Protein targeting to various cellular compartments were also evaluated to demonstrate strain capabilities.

I have two related recommendations to provide the authors. The title of the manuscript states the objective of the work is "platform" development for recombinant protein production. That implies an entire production system from strain improvement to scaled up cultivation. Yet the work that is described covers gene design through phenotyping at a scale that is not described. Hence, recommendation one is to add detail to the cultivation section of the M&M to include volumes cultivated and culture conditions used; that would include sterility requirements. Second, please describe the intended cultivation platform that leads to economic viability. Would that involve raceway ponds, photobioreactors - and what type of each would be compatible with the strains described. What are the maximum culture volumes have been achieved with Chlamydomonas recombinant strains. All those details will be of great interest to those considering adoption and could easily be provided in the introduction or M&M sections. Alternative, the authors could elect to change the title to reflect the strain engineering focus of the work without reference to platform development.

Authors revisions:

- **Recommendation 1:** We modified the text under "Growth conditions" in the Materials and methods section: "Strains were cultivated in 50 mL of TAP media at 25°C with constant illumination at 80 μmol photons $m^{-2}s^{-1}$ and agitated at 150 rpm on a rotary shaker."
- **Recommendation 2:** We elected to change the title to "Engineering the green algae *Chlamydomonas incerta* for recombinant protein production" to reflect the strain engineering focus of our work.

---

6. PLOS authors have the option to publish the peer review history of their article (what does this mean?). If published, this will include your full peer review and any attached files.

If you choose "no", your identity will remain anonymous but your review may still be made public.

Do you want your identity to be public for this peer review? For information about this choice, including consent withdrawal, please see our Privacy Policy.

Reviewer #1: No
